# The Prevalence of Bacterial Vaginosis in Pregnant Women in Slovenia, Determined via Microscopy and Semi-Quantitative Relative Culture, and Its Association with Adverse Pregnancy Outcomes

**DOI:** 10.3390/microorganisms13030588

**Published:** 2025-03-04

**Authors:** Maja Starc, Miha Lučovnik, Petra Eržen Vrlič, Samo Jeverica

**Affiliations:** 1 National Institute of Public Health, 1000 Ljubljana, Slovenia; maja.starc@nijz.si; 2 Department of Perinatology, University Medical Centre Ljubljana, 1000 Ljubljana, Slovenia; miha.lucovnik@kclj.si; 3 Medical Faculty, University of Ljubljana, 1000 Ljubljana, Slovenia; 4 Department of Obstetrics and Gynecology, Community Health Center Ljubljana, 1000 Ljubljana, Slovenia; petra.erzen-vrlic@zd-lj.si; 5 Izola General Hospital, 6310 Izola, Slovenia; 6 Faculty of Health Sciences, University of Primorska, 6000 Koper, Slovenia

**Keywords:** bacterial vaginosis, culture, microscopy, pregnancy, preterm birth, preterm premature rupture of membranes, Slovenia

## Abstract

Bacterial vaginosis (BV) is associated with various adverse pregnancy outcomes. It is usually diagnosed via microscopy. Semi-quantitative relative culture (SRC) was investigated as a complementary diagnostic method to determine the prevalence of BV and its association with preterm birth and preterm, premature rupture of membranes (PPROM) in pregnant women in Slovenia. We examined 3437 consecutive vaginal swabs from pregnant women during the five-year period and were able to link the results to 2531 pregnancy outcomes. The isolates were identified using MALDI-TOF, and the results were assessed by the relative amounts of *Gardnerella vaginalis* and lactobacilli according to two stringency criteria. The prevalence of BV was 6.5% via microscopy and was higher for SRC, 9.9% or 11.1%, depending on the stringency criteria. The association with adverse pregnancy outcomes was better when SRC was used, resulting in adjusted odds ratios of 1.76 (1.30 to 2.37) and 1.97 (1.38 to 2.82) for preterm birth and PPROM, respectively, with more stringent interpretation. Microscopically detected BV was not associated with either outcome. The clinical validity of SRC was demonstrated by its better correlation with adverse pregnancy outcomes in a large cohort of pregnant women. SRC with MALDI-TOF identification is a promising advancement of vaginal culture.

## 1. Introduction

Bacterial vaginosis (BV) is the most common disease of the lower genital tract in women of childbearing age and the most common cause of vaginal discharge during pregnancy [1,2,3]. It is characterized by a shift in the bacterial microbiota from a predominance of lactobacilli, particularly *Lactobacillus crispatus*, to a predominance of mixed aerobic and anaerobic bacteria, particularly *Gardnerella vaginalis*, *Atopobium vaginae* and *Prevotella bivia* [4,5,6]. Bacterial vaginosis is associated with various adverse pregnancy outcomes, particularly preterm birth and preterm, premature rupture of membranes (PPROM), as well as other conditions, such as chorioamnionitis, maternal postpartum infections and an increased risk of neonatal sepsis [7,8,9].

The diagnostic evaluation of BV remains suboptimal, with misdiagnosis and inappropriate treatment occurring in up to 40% of cases [10]. Several factors contribute to this fact. First, approximately 50% of cases are asymptomatic, reducing clinical suspicion and leading to underdiagnosis. In addition, diagnostic protocols rely heavily on clinical assessment using the Amsel criteria (i.e., three of four clinical signs present; (i) typical discharge, (ii) “clue” cells on microscopy, (iii) positive whiff test and (iv) pH > 4.5) [11], a method with well-documented limitations in terms of sensitivity and specificity, which contributes to diagnostic inaccuracies [10,12]. Finally, microscopic evaluation of Gram-stained vaginal swabs, mostly using the Nugent scoring system (i.e., scoring based on the quantification of three morphotypes of bacteria—(i) lactobacilli, (ii) gardnerella and (iii) mobilluncus morphotype—and defines three vaginal flora states: normal, intermediate and BV), is still considered the gold standard for BV diagnosis [13,14]. This is despite the fact that bacterial differentiation via microscopy is based on only two bacterial parameters, morphology and staining characteristics, and these are insufficient to distinguish the four major species of vaginal lactobacilli (*L. crispatus*, *L. iners*, *L. jensenii* and *L. gasseri*) and to define the increasingly complex mixed bacterial populations seen in BV [4,13]. Given the recent developments in culture-based diagnostics, in particular the easier and more reliable identification of bacteria using MALDI-TOF systems, but also the use of specialized media and the ability to (semi-)quantify the bacteria present in the sample, many speak of a revival of culture [15]. It is a comprehensive method that can identify a large proportion of the microbial causes of vaginitis, including those not currently included in the available molecular syndromic panels [16]. Nevertheless, there are no studies that evaluate the semi-quantitative relative culture (SRC) of vaginal swabs for the diagnosis of BV and provide comprehensive identification of all predominant microorganisms in the sample, including lactobacilli, *G. vaginalis*, aerobic vaginitis-associated bacteria, yeasts and others.

The prevalence of BV in pregnancy varies in different populations and ranges from 6% to 32% [3,7,8]. No such data are available in Slovenia. The primary aim of this study was to determine the prevalence of BV in pregnant women in Slovenia using both microscopy and SRC. The secondary aim was to determine the association of BV during pregnancy, as determined via the two diagnostic methods, with two major adverse pregnancy outcomes, preterm birth and PPROM, in our cohort. We hypothesize that the prevalence of BV vary based on the diagnostic method used and that we will be able to demonstrate an association between BV, preterm birth and PPROM.

## 2. Materials and Methods

### 2.1. Study Population

This was a retrospective cohort study involving two obstetrics and gynecology centers in the Central Slovenia region, which serve approximately 20% of pregnant women nationwide, namely the University Medical Center (UMC) Ljubljana and the Community Health Center (CHC) Ljubljana. We analyzed the microbiological results of vaginal swabs taken from asymptomatic and symptomatic pregnant women from 2014 to 2018. A single swab per pregnancy (i.e., the first) was included in the analysis. Swabs collected for screening for Group B Streptococcus (GBS) were excluded from the analysis. The microbiological data were linked to data from the National Perinatal Information System (NPIS). Since 1987, all births in Slovenia at the 22nd completed week of gestation or later or when the neonatal birth weight is ≥500 g have been registered in the NPIS. Registration is required by law in all maternity wards in the country, and more than 140 variables are entered into a computerized database by the attending midwife and doctor. The patient’s demographic data, family, medical, gynecological and obstetric histories, data on the current pregnancy, labor and birth, the postpartum period and neonatal data are recorded. The calculation of gestational age in the NPIS is based on the last menstruation or on the ultrasound estimate (calculated using the crown–rump length measured in the first trimester) if this differs by ≥7 days.

### 2.2. Microbiological Methods

Vaginal swabs with Amies transport medium were used in this study. They were transported to the laboratory at room temperature within 24 h of collection. SRC was performed with inoculation in 4 quadrants. The swabs were inoculated onto 5 agar media: blood agar (BA), chocolate agar (CA), colistin–nalidixic acid agar (CNA), ChromID Strepto B (STRB; bioMérieux, Marcy l’Etoile, France) and *Gardnerella vaginalis* Selective Agar with human blood (GARD; Oxoid, Basingstoke, UK). Finally, the swabs were mounted on a slide for Gram staining and microscopy. The plates were incubated in a normal atmosphere (BA, CNA and STRB) and in a 5% CO_2_-enriched atmosphere (CA and GARD) and examined for the presence of growth after 24 and 48 h. All typical colonies were identified using the MALDI biotyper (Bruker Daltonics, Hamburg, Germany). The quantity of growth was determined semi-quantitatively for each species as 1+, 2+ or 3+ if the bacteria grew in the first, second or third/fourth quadrant, respectively.

### 2.3. Detection of BV via Microscopy

Gram-stained smears were examined for the presence of BV using the Nugent score. Briefly, the quantity of morphotypes of lactobacilli, *Gardnerella* and *Mobiluncus* was determined and converted into discrete scores ranging from 0 to 4 for lactobacilli and *Gardnerella* and from 0 to 2 for *Mobiluncus*. Final scores from 0 to 10 were obtained, which were interpreted as normal flora (0–3), intermediate flora (4–6) and BV (7–10) [17].

### 2.4. Detection of BV via SRC

**Figure 1:**
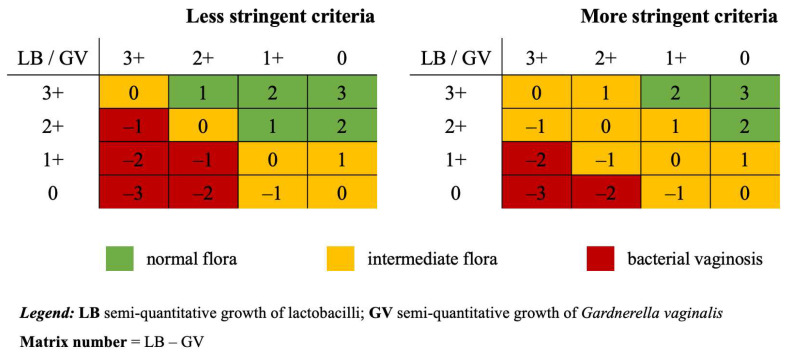
Semi-quantitative relative culture (SRC) interpretation criteria. See text for a detailed explanation of the less stringent criteria and the more stringent criteria.

The growths of lactobacilli and *G. vaginalis* were recorded on CA and GARD agar plates after 48 h of incubation in an atmosphere enriched with 5% CO_2_. In brief, CA is a general-purpose agar for the isolation of fastidious microorganisms and contains a GC agar base, bovine hemoglobin and IsoVitaleX enrichment. GARD is a specialty agar for the detection of *G. vaginalis* and contains blood agar base, human erythrocytes for the detection of hemolysis, Tween 80 to enhance hemolysis size and gentamicin, nalidixic acid and amphotericin B to selectively inhibit commensals and yeasts. Culture results were interpreted using 3 categories (i.e., normal, intermediate and BV) and 2 stringency criteria (i.e., less and more stringent): (i) normal flora was defined if lactobacilli were present at a level ≥2+ and the relative difference to *G. vaginalis* was ≥1+ (less stringent) or ≥2+ (more stringent); (ii) BV was defined if *G. vaginalis* ≥2+ was present and the difference with lactobacilli was ≥1+ (less stringent) or ≥2+ (more stringent); (iii) an intermediate flora was defined when the relative difference between lactobacilli and *G. vaginalis* was from −1 to 1 (more stringent) and, additionally, the amount of both groups was ≤1+ (less stringent) (Figure 1). If the results of the microscopy and culture categories differed, both were reassessed by a second observer, and these results were considered final.

### 2.5. Data Management and Statistical Analysis

Both datasets, i.e., the microbiology dataset and the NPIS dataset, were quality assured, combined using a unique identifier and manually corrected for the presence of duplicate values due to multiple pregnancies, multiparous pregnancies and multiple swabs taken during a pregnancy. A single datasheet with non-duplicate entries of anonymized demographic data, microbiological data and pregnancy outcome data (i.e., preterm birth, PPROM and confounding factors) was created and used for statistical analysis. For statistical analysis, descriptive statistics, prevalence estimates, chi-square tests to compare proportions between independent groups and logistic regressions were calculated using the open-source statistical package JASP 0.19. Data were presented with proportions and 95% confidence intervals, and a *p*-value of <0.05 was considered statistically significant.

## 3. Results

### 3.1. Study Population

A total of 3437 consecutive vaginal swabs (i.e., one per pregnancy) from 3284 pregnant women were analyzed. Of these, 141 were from two pregnancies and 6 from three pregnancies. The swabs were obtained in the amount of 77.2% (n = 2655) at the UMC Ljubljana and 22.8% (n = 782) at the CHC Ljubljana. The mean age of pregnant women at the time of swab collection was 31 years (14–52 years). The annual distribution of swabs increased steadily during the study period, ranging from 604 swabs in 2014 to 809 swabs in 2018. The median gestational age at swab collection was 28 weeks (4–41 weeks). The distribution of swabs was skewed towards a higher gestational age with a mode of 32 weeks. In 2531 pregnancies (73.6%), we were able to clearly match the microbiological results with the pregnancy outcome data.

### 3.2. Microbiological Characteristics

The microbiological isolates are listed in Table 1. Lactobacilli were the most frequently isolated and identified microorganisms in our study. A total of 2497 lactobacilli species were isolated, mostly a single species per swab, while 2 lactobacilli species were isolated in 28 swabs. At least one *Lactobacillus* species was detected in 71.8% (n = 2469) of the samples, most frequently *L. crispatus* in 27.1% (n = 931) of the samples, followed by *L. jensenii* (14.3%, n = 491), *L. gasseri* (13%, n = 448), *L. iners* (10.5%, n = 361) and other lactobacilli as a group (7.7%, n = 266), most of which were the known probiotic species *L. delbrueckii* and *L. rhamnosus*. *G. vaginalis* was culture positive in 16.8% (n = 577) of samples and was detected at a high concentration in 83.9% (n = 484) of samples using the semi-quantitative method of streaking (3+ growth). Strong growth (2+ or 3+) of bacteria from the mixed aerobic bacteria group was isolated in 20.1% (n = 691) of the samples. This group consisted of members of the Enterobacterales, enterococci, GBS and *S. anginosus*. Yeasts were detected in 20.3% (n = 696) of the samples, most of them at high concentrations, either 3+ (8.3%; n = 286) or 2+ (8.3%; n = 284), while GBS without enrichment was detected in 14.2% (n = 489) of the vaginal swabs. Combined vaginal and rectal swabs were not analyzed in this study.

### 3.3. Prevalence of BV

The prevalence of BV as determined via microscopy and SRC is shown in Table 2. Overall, 76.7% (n = 2637) of the samples had a Nugent score of 0–3, corresponding to normal flora; intermediate flora (Nugent score 4–6) was present in 16.8% (n = 576) of samples; and BV (Nugent score 7–10) was present in 6.5% (n = 224) of samples. Based on SRC, BV and intermediate flora were detected in 11.1% (n = 380) and 23.5% (n = 807), respectively, with less stringent interpretation and in 9.9% (n = 341) and 25.4% (n = 874), respectively, with more stringent interpretation. Normal flora was detected in 65.6% (n = 2250) and 64.6% (n = 2222) of the samples with less stringent and more stringent interpretations, respectively. The correlation between the results of microscopy and SRC for both categories of BV and other microorganisms detected in culture is shown in Table 3.

*Lactobacillus crispatus* and *L. jensenii* were significantly more abundant in cultures without the simultaneous presence of G. vaginalis (*p* < 0.001 for both). A negative correlation was not observed for *L. gasseri* (*p* = 0.433) and *L. iners* (*p* = 0.835). Yeasts (*p* = 0.002) and bacteria associated with aerobic vaginitis (*p* < 0.001) were also negatively correlated with the presence of *G. vaginalis*.

### 3.4. Adverse Pregnancy Outcomes and BV

The correlation between adverse pregnancy outcomes and categorical microscopy and SRC results (i.e., normal, intermediate and BV) is shown in Table 4. Odds ratios were adjusted for maternal age, smoking during pregnancy and multiplicity. Interestingly, the intermediate category (Nugent score 4–6) determined via microscopy was a stronger predictor (risk factor) of both preterm birth and PPROM, with adjusted odds ratios of 2.53 and 3.28, respectively, while the BV category (Nugent score 7–10) was not a significant predictor. Both the “intermediate” category and the “bacterial vaginosis” category, as determined via SRC, significantly predicted preterm birth and PPROM. A less stringent interpretation of culture was a slightly better predictor of pregnancy outcome (Table 4).

## 4. Discussion

In this large study, which spanned a 5-year period and included almost 3500 vaginal swabs from pregnant women in Slovenia evaluated via microscopy, the current gold standard for BV detection, as well as comprehensive SRC combined with the identification of all predominant bacterial species using MALDI-TOF mass spectrometry, we observed a significant difference in the prevalence of BV determined using the two methods and interpretation criteria. The prevalence determined via microscopy was 6.5%, while the prevalence determined via SRC was 9.9% and 11.1%, depending on the stringency criteria used to interpret the cultures.

In a subgroup analysis of more than 2500 pregnancies with known outcomes, we tested the categorical results of both methods of detecting BV as predictors of preterm birth and PPROM. We found that the intermediate category determined using microscopy was a better predictor of both adverse outcomes, whereas BV based on microscopy was not a significant predictor. Both categories, determined via SRC and interpreted according to either less stringent or more stringent interpretation, were significant predictors of the observed adverse pregnancy outcomes.

Preterm birth is the leading cause of perinatal morbidity and mortality worldwide, and although it is multifactorial, it has been clearly associated with BV [3,9,18,19]. When BV is diagnosed during pregnancy, the risk of preterm birth increases more than twofold, and this association is even more pronounced when the diagnosis is made earlier in pregnancy, before 20 weeks of gestation [20]. However, intervention studies of antimicrobial treatment of BV during pregnancy to prevent preterm birth have produced equivocal results. Earlier studies conducted in a high-risk population have shown that antimicrobial treatment reduces the incidence of recurrent preterm birth by two-thirds [21], while later studies conducted in the general pregnant population have been less encouraging and have shown no such beneficial effect [8,22]. These findings are reflected in most current recommendations, which advocate the treatment of symptomatic BV during pregnancy but do not recommend screening or treating asymptomatic women without a history of preterm birth [7,23,24]. The discrepancy between association and intervention studies may be due to the fact that the causal relationship between BV and preterm birth has not been recognized. However, it may also be due to inaccuracies in the diagnostic tests. We have shown here that microscopy, the gold standard for the detection of BV, greatly underestimates the prevalence, as evidenced by the strong growth of *G. vaginalis* and the concomitant low or absent growth of protective lactobacilli, as determined via SRC. Our results suggest that the diagnosis of BV is strongly influenced by the methodology used. The majority of the discordant culture-positive BV samples belonged to the “intermediate” category, as determined via microscopy. Interestingly, in two intervention studies that included women with abnormal vaginal flora (i.e., BV and intermediate flora), treatment with antibiotics reduced the risk of preterm birth by almost half (OR = 0.53) [25,26].

In addition to the conventional method of detecting BV (microscopy), we have also used a less conventional but classical approach (culture). This approach underwent a major improvement in recent years with the development of reliable identification using MALDI-TOF mass spectrometry, which allowed us to easily identify all predominant bacteria growing on agar plates. With better identification, the focus of the culture shifted to determining the five microbiota types rather than just looking for pathogenic bacteria [4]. We were able to identify all four major species of lactobacilli and were even able to show that in the case of *L. iners* and *L. gasseri*, the protective effect against colonization and overgrowth by *G. vaginalis* was diminished, as these two were frequently detected in combination with *G. vaginalis*, in contrast to the two protective lactobacilli, *L. crispatus* and *L. jensenii* [27]. We used two agar plates for the isolation of *G. vaginalis*, namely CA and GARD agar. Although *G. vaginalis* can grow well on CA, it has previously been shown that isolation is improved by using a selective and differential medium such as GARD [11]. Furthermore, it is known from previous studies that the mere presence of *G. vaginalis* in culture is not indicative of BV, as it can also colonize healthy vaginal mucosa. Here, we introduced SRC, in which we compared the amount of growth of *G. vaginalis* and lactobacilli when both were simultaneously present in culture by subtracting their amounts (lactobacilli—*G. vaginalis*). The same principle was used in a previous study and improved the predictive value of the culture [28]. As a rule of thumb, the presence of 3+, 2+ and 1+ growths corresponds to concentrations of approximately 10^6^, 10^5^ and 10^4^ CFU/mL, respectively. In accordance with this approximation, the difference from 3+ to 1+ represents a 100-fold difference in concentration.

In Slovenia, there are no data on the prevalence of BV in the general population or in pregnant women. Our results are consistent with those of previous studies conducted in the same geographical region and with the same method (microscopy), in which the prevalence ranged from 5 to 12% [25,29,30,31]. A higher prevalence of around 15–30% was found in US and African studies [3,18,32,33]. In addition, the method of diagnosis may influence the prevalence of BV, as was also found in our study [34,35]. Several other studies that used a different diagnostic approach, particularly Pap smear analysis, observed a higher prevalence [35,36]. Other factors that may influence prevalence include ethnicity, socioeconomic status, smoking, age, body mass index, sexual practices and possibly other factors [8,30].

Finally, we were able to combine our microbiology dataset with the clinical outcomes dataset (NPIS) to test the clinical validity of microscopy and SRC in predicting two adverse pregnancy outcomes. Surprisingly, the “intermediate” category rather than the “bacterial vaginosis” category, as determined by the Nugent score, was a much better predictor of both preterm birth and PPROM. When the culture results were tested to predict pregnancy outcomes, both “non-normal” categories were found to be significant, regardless of which stringency criterion was used for interpretation. The risk for both adverse outcomes increased approximately twofold with each category (“intermediate” and “bacterial vaginosis”).

Our study has several limitations. First, it was a retrospective study, and although we used both microscopy and SRC, the Amsel criteria are not usually performed in our country, as is common elsewhere. Secondly, we have no information on the indication for swab collection. Thus, our cohort represents a mixture of women with symptomatic vaginitis and asymptomatic cases. However, symptomatic BV occurs in only about half of affected pregnant women, so symptoms may be unreliable for the detection of vaginal dysbiosis. Third, we were not able to link all swab results to pregnancy outcomes because the database was sometimes incomplete. Nevertheless, we were able to link a large majority of the swab results to clinical outcomes, which makes our dataset valuable for further analysis of the relationship between microbiology and clinical outcome. Finally, the major advantage of our study is the comprehensive culture, with the identification of all microorganisms, including lactobacilli, an approach similar to the determination of vaginal microbiota using other, mainly molecular, methods.

Considering all the developments in diagnostic methods in clinical microbiology, it is quite surprising that microscopy is still the gold standard for the detection of BV, since it is not possible to fully distinguish the bacilli observed under a microscope and since microscopy is the least sensitive bacteriological method. Here, we have shown that the morphotypes of both lactobacilli and *G. vaginalis* can be more reliably determined using standard culture and identification methods. The latter method in particular has evolved over the last ten years and allows us to reliably identify virtually all bacteria grown on agar plates. In addition, this approach allows us to apply the principle of relative abundance, as used in the Nugent score, with much greater accuracy and identify other common vaginitis pathogens (i.e., yeasts, aerobic vaginitis-associated bacteria, streptococci, staphylococci, etc.). As most of the currently available molecular methods for the detection of BV are not comprehensive and do not identify all possible vaginitis pathogens, culture remains a very important method that requires further methodological and interpretative development. Further studies are needed to validate our findings and possibly re-evaluate the potential therapeutic intervention of BV during pregnancy in terms of reducing the risk of adverse pregnancy outcomes.

## 5. Conclusions

In summary, the prevalence of bacterial vaginosis in pregnant women in our cohort was highly dependent on the diagnostic method used. Microscopy according to the Nugent criteria underestimated the prevalence by several points compared to SRC. The clinical validity of culture was demonstrated by its correlation with adverse pregnancy outcomes. Further studies are needed to validate our results, but microscopy is probably not accurate enough to serve as a gold standard for the microbiologic diagnosis of BV.

## Figures and Tables

**Table 1:** Basic demographic and microbiological characteristic.

Parameter	n	% (Isolates)	% (Swabs/Pregnancies)
Demographic characteristics			
Pregnant women	3284	/	95.5
Swabs/pregnancies	3437	/	100
Pregnant women age (mean, range)	31 (14–51)	/	/
Gestational age (median, range)	28 (4–41)	/	/
Microbiological isolates * (n = 4922)			
Lactobacilli	2497	50.9	§
*Lactobacillus crispatus*	931	18.8	27.1
*Lactobacillus jensenii*	491	9.9	14.3
*Lactobacillus gasseri*	448	9.1	13.0
*Lactobacillus iners*	361	7.3	10.5
Other lactobacilli	266	5.8	7.7
Gardnerella vaginalis	577	11.7	16.8
Mixed aerobic bacteria	1152	23.3	§
*Streptococcus agalactiae*	489	9.9	14.2
*Enterococcus faecalis*	178	3.6	5.2
*Streptococcus anginosus*	171	3.5	5.0
*Escherichia coli*	140	2.8	4.1
*Staphylococcus aureus*	61	1.2	1.8
Others	113	2.3	§
Yeast	696	14.1	20.3

* More than one bacterial isolate could be isolated from a single vaginal swab. The cumulative proportion of isolates is shown in the table and does not correspond to the incidence of bacterial vaginosis. § Mixed group of microorganisms: the proportion among swabs cannot be determined, as more than one/group possible may have a footer.

**Table 2:** Prevalence of bacterial vaginosis (BV) based on different diagnostic method and interpretation criteria.

Diagnostic Method/Vaginal Microbiota Category	n	% (Swabs/Pregnancies)
Microscopy (Nugent score)		
Normal (0–3)	2637	76.7
Intermediate (4–6)	576	16.8
Bacterial vaginosis (7–10)	224	6.5
Total	3437	100
SRC ^#^ (less stringent criteria)		
Normal	2250	65.5
Intermediate	807	23.5
Bacterial vaginosis	380	11.1
Total	3437	100
SRC ^#^ (more stringent criteria)		
Normal	2222	64.6
Intermediate	874	25.4
Bacterial vaginosis	341	9.9
Total	3437	100

^#^ SRC: semi-quantitative relative culture including only BV-associated cultured microorganisms (*Gardnerella vaginalis* and *Lactobacillus* spp.).

**Table 3:** Correlation between vaginal microbiota categories using microscopy and semi-quantitative relative culture.

Vaginal Microbiota Categories	SRC ^#^ (Less Stringent Criteria)				SRC ^#^ (More Stringent Criteria)			
Microscopy (Nugent Score)	Normal	Intermediate	BV *	Sum	Normal	Intermediate	BV *	Sum
Normal (0–3)	2174	414	49	2637	2152	448	37	2637
Intermediate (4–6)	71	372	133	576	68	394	114	576
BV * (7–10)	5	21	198	224	2	32	190	224
Sum	2250	807	380	3437	2222	874	341	3437

^#^ SRC: semi-quantitative relative culture including only BV-associated cultured microorganisms (*Gardnerella vaginalis* and *Lactobacillus* spp.). * BV: bacterial vaginosis.

**Table 4:** Correlation between adverse pregnancy outcomes and bacterial vaginosis (BV) as determined using microscopy and semi-quantitative relative culture.

Pregnancy Outcome	Odds Ratio (95% Confidence Interval)			
Diagnostic Method/Criteria	Crude	*p*-Value	Adjusted *	*p*-Value
Preterm Birth				
Microscopy (Nugent score)				
Normal	0.38 (0.35 to 0.42)	<0.001	0.28 (0.25 to 0.31)	<0.001
Intermediate	2.41 (1.94 to 2.99)	<0.001	2.53 (2.01 to 3.178)	<0.001
Bacterial vaginosis	0.87 (0.60 to 1.26)	0.458	0.87 (0.58 to 1.29)	0.487
SRC ^#^ (less stringent criteria)				
Normal	0.36 (0.32 to 0.40)	<0.001	0.26 (0.23 to 0.29)	<0.001
Intermediate	1.89 (1.56 to 2.30)	<0.001	1.99 (1.61 to 2.45)	<0.001
Bacterial vaginosis	1.65 (1.26 to 2.17)	<0.001	1.72 (1.29 to 2.29)	<0.001
SRC ^#^ (more stringent criteria)				
Normal	0.36 (0.32–0.40)	<0.001	0.26 (0.23 to 0.29)	<0.001
Intermediate	1.77 (1.46–2.15)	<0.001	1.88 (1.53 to 2.31)	<0.001
Bacterial vaginosis	1.70 (1.28–2.26)	<0.001	1.76 (1.30 to 2.37)	<0.001
PPROM ^†^				
Microscopy (Nugent score)				
Normal	0.11 (0.09 to 0.13)	<0.001	0.09 (0.08 to 0.11)	<0.001
Intermediate	3.21 (2.46 to 4.19)	<0.001	3.28 (2.55 to 4.23)	<0.001
Bacterial vaginosis	0.75 (0.41 to 1.38)	0.356	0.84 (0.48 to 1.46)	0.533
SRC ^#^ (less stringent criteria)				
Normal	0.10 (0.09 to 0.12)	<0.001	0.09 (0.07 to 0.10)	<0.001
Intermediate	2.24 (1.72 to 2.92)	<0.001	2.24 (1.74 to 2.87)	<0.001
Bacterial vaginosis	1.89 (1.31 to 2.72)	<0.001	1.99 (1.41 to 2.80)	<0.001
SRC ^#^ (more stringent criteria)				
Normal	0.10 (0.09 to 0.12)	<0.001	0.09 (0.07 to 0.10)	<0.001
Intermediate	2.10 (1.61 to 2.72)	<0.001	2.14 (1.67 to 2.74)	<0.001
Bacterial vaginosis	1.91 (1.30 to 2.80)	<0.001	1.97 (1.38 to 2.82)	<0.001

^#^ SRC: semi-quantitative relative culture including only BV-associated cultured microorganisms (*Gardnerella vaginalis* and *Lactobacillus* spp.). ^†^ PPROM: preterm premature rupture of membranes. * Adjusted for maternal age, smoking during pregnancy and multiplicity.

## Data Availability

The data presented in this study are available on request from the corresponding author. The data are not publicly available due to ethical restrictions.

## Abbreviations

The following abbreviations are used in this manuscript:

BV	Bacterial vaginosis
GBS	Group B streptococcus
GV	Gardnerella vaginalis
LB	Lactobacilli
MALDI-TOF	Matrix-assisted laser desorption ionization–time-of-flight mass spectrometry
NPIS	National Perinatal Information System
PPROM	Preterm, premature rupture of membranes
SRC	Semi-quantitative relative culture
